# Surgical management of extensive burns treatment using allografts

**Published:** 2012-12-25

**Authors:** DR Calota, C Nitescu, IP Florescu, I Lascar

**Affiliations:** * Emergency Hospital for Plastic, Reconstructive and Burns Surgery, Bucharest; ** "Bagdasar Arseni" Emergency Hospital, Bucharest; *** "Floreasca" Emergency Hospital, Bucharest "Carol Davila" University of Medicine and Pharmacy, Bucharest

**Keywords:** allotransplant, skin graft, allograft, postcombustional sequelae

## Abstract

Patients with extensive burns (TBSA over 45%) can benefit from treatment with split thickness skin allotransplants (skin bank or honorific donors).

In this study, we present our protocols for the surgical treatment by using allografts. We emphasize the criteria for the staging of the procedures, the prioritisation of the areas that need to be grafted and the postoperative management. The treatment includes a serial excision grafting with simple grafts or the sandwich method, which implies the covering of the wound with a widely meshed autograft (6:1). This layer is covered by a 1,5:1 or 3:1 expanded mesh allograft.

## Introduction

The pathology of extensive burns is a very complex one, affecting the homeostasis of the organism at the level of all the organs and systems, with complications that often lead to death. The survival rate increased by means of improving the treatment of extensive burns with a total body surface area (TBSA) > 25%, so that, among our clinical cases, we even had a patient who survived a 95% TBSA burn. This treatment is very expensive, involves material and human resources, the patients are hospitalised usually for a period of over 60-80 days (an average of 1 day for each percent of burned skin). This is followed by several years of treatment of the postcombustional sequelae. Rehabilitation and adapting to this new life is very difficult for the patient, who needs a great will power and time.

Wound covering for burns can be done with temporary bioactive skin substitutes.

Temporary bioactive skin substitutes:

-Human allografts taken from cadaver and kept in a skin bank, or from honorific donors. This provides coverage with both dermis and epidermis

-Porcine xenografts bioactive dermal-like matrices that provide only the epidermal layer

-Amniotic membrane 

-Biobrane double layer dressing with silicone on the outside and nylon with collagen on the inside

-Transcyte double layer dressing with silicone on the outside and nylon seeded with neonatal fibroblasts on the inside. 

The advent of treating burns by early excision and grafting in the late 19th century brought up the need of finding a temporary wound cover before the more shallow burns have time to heal, in order to provide skin for an autograft. Allografts or homografts can be taken either from live donors or in the form of cadaveric skin that is usually preserved in a skin bank. The first reported use of cadaveric skin to cover a burn wound was in 1881. This might also be the first reported case of possible rejection: termed erysipelatous inflammation occurred and the graft was lost in the second week [**1**].

## Materials and methods

This study retrospectively followed 148 patients with extensive burns of over 25% between 2002-2009, of whom 76 received treatment with allografts. Moreover, during 2010-2011, 47 new patients enrolled, of whom 13 were treated with allografts.

After the burn event, the patient had to be transported to a specialized unit as fast as possible with great care, while respecting the measures of asepsis.

Intensive Care Unit (I.C.U.) management of extensive burns (over 25% TBSA)

**Fluid Resuscitation**

Crystalloids are the main solution used for fluid resuscitation (Natrium chloride and Lactated Ringer's solution). The volumes required vary greatly, with a mean value of about 4 ml per kilogram multiplied by the percentage of body-surface area burned. Fluid requirements are greater if resuscitation has been delayed and in patients with smoke inhalation. Large volumes are needed because only 20 to 30 percent of the crystalloid administered remains with the vascular system. About half the fluid must be given during the first eight hours after the injury, in the same period of rapid edema formation. In most patients, the hourly rate of urine flow, a reasonable indicator of organ perfusion, is the principal guide used to alter the rate of fluid administration (about 1ml/min=60 ml/hour). Abrupt weight gains of 30 percent or more of the body weight can result from resuscitation in patients with massive injury; early endotracheal intubation and multiple limb and truncal escharotomies are commonly required in these circumstances. Pulmonary-artery catheters are not routinely used but are helpful in elderly patients and in those with limited cardiac reserves. Survival after near-total burns is no longer uncommon, provided that early organ failure is avoided [**17**]. The crystalloid requirement during the second day of treatment is about half that of the first day. Within 48 to 72 hours after the burn injury, the hematocrit begins a progressive fall due to factors such as intravascular resorption of edema, lysis of thermally injured cells, and the onset of the anaemia that is characteristic after a burn injury. Crystalloid administration should be discontinued at the earliest possible time. A reasonable goal is for the patient to have returned to his or her pre-injury weight within one week after the burn [**17**].

Resuscitation by using fluids containing solutes with plasma-like oncotic properties has an intuitive appeal, since these fluids resemble what is being lost and protein sieving is still at least partially operative in unburned tissues. Nevertheless, the adjunctive use of colloid in burn shock has decreased, mainly because the controlled trials that have been done have shown no clear advantage to its use [**17**].

Colloid administration for 12 to 24 hours after the injury (use of HES solution -4 mg/kg body weight) — a time when capillary permeability has partially returned to normal but plasma volume may be subnormal. This practice has no demonstrated clinical benefit, however, and recent data suggest that it may be deleterious. The administration of albumin to patients in stable condition after 24 hours of clinically satisfactory crystalloid resuscitation resulted in a significant decrease in the glomerular filtration rate, below the normal range, despite an increase in plasma volume [**17**].

The administration of crystalloid with a sodium concentration of 250 mmol per litre can reduce volume requirements, presumably by mobilizing water from cells that are over-hydrated as a consequence of the injury [**17**]. This therapy is useful in patients with limited cardiopulmonary reserves but demands careful monitoring and clearly has a much narrower therapeutic margin than does the use of isotonic crystalloid [**17**]. Systemic antimicrobial therapy is not mandatory from the start (it is used only if the antibiogram shows the presence of microbial cultures or if there are increased values of fibrinogen or leukocytosis); early antimicrobial therapy does not influence the prognosis. We insist on prophylactic measures on venous, arterial and urine catheters.

The algorithm for intensive burn resuscitation also includes the assurance of the function of the enterocyte through early enteral nutrition.

**Surgical treatment of severe burns **

At the admission: adequate local debridement with Betadine scrub or Chlorhexidine solutions; also decompression incisions (where these are required); dressing with Flamazine or Flammacerium (the last one, applied in the first post burn hours, is blocking the release of lipoproteins responsible for the cytokines storm leading to SIRS).

The surgical treatment can be considered after the patient has surpassed the acute stage, usually after 3-5 days, and is done either by early excision and grafting or by assisted detersion, which aims to obtain a granular wound surface that will eventually lead to healing.

For deep burns that are too extensive to be closed in one procedure, wound excisions can be staged — typically at intervals of about one week — as sufficient autologous skin grafts become available to close the excised wound. Alternatively, the burns can be completely excised within the first several days after the injury, and a temporary skin substitute used to close the wound remaining after all available autologous skin has been harvested and grafted. 

We have to establish a prioritisation of the areas that we want o excise. First, we have to think about the functional areas such as the hands, and then the 3rd degree burns of the thorax, where the allograft has a better chance. For the extensive burns, we often have to consider the use of allografts at this stage, because there is a lack of uninjured skin that could be used for an autograft. 

Fresh or cryopreserved allogeneic skin from cadavers is the most reliable wound cover, although its use has a small risk of disease transmission.

In our clinic, we used skin from honorific donors who were usually relatives or friends of the patient. It was of paramount importance to obtain complete and accurate medical information about the potential donor to ensure the safety of the tissue for transplantation). This required a comprehensive medical and social history of the donor, a physical examination of the potential donor, and a panel of serologic screening tests for viral diseases (HIV-1/2, Hepatitis B and C). It was imperative that skin banks performed microbial cultures before the release for transplantation.

The donors also had to sign a specific informed consent by which they agreed to donate skin, knowing the risks of donating organs and/or tissues/cells, according to the legal status for these procedures, understanding this gesture as being purely humanitarian, without being pressured in any way or expecting a material reward. Split-thickness skin grafts were removed by using an electric dermatome with a thickness of 0.012 to 0.018 inches. 

We applied the allograft as a simple graft meshed 1,5:1 or 3:1 or as a sandwich graft which implied the covering of the wound with a widely meshed autograft (6:1). This layer was covered with a 1,5:1 or 3:1 expanded mesh allograft.

Another common use of the allograft was to test a questionable wound bed, in excisions that were carried down near tendons, bone, or fascia of questionable viability. If the allograft needed it, the wound bed usually will also take an autograft [**2**].

The allograft was rejected in an interval of time that varied between 14-21 days, but in this period of time the more shallow burns will heal and provide material for autograft.

In these situations, we had to use every resource of skin that was available, so that we had to consider taking skin from unusual sites as the soles of the feet or the scalp.

## Results

In this case, we treated a young patient with a 95% TBSA burn, IIA, II B and III degree (of which 60% were III degree burns).

**Fig. 1 a,b,c:**
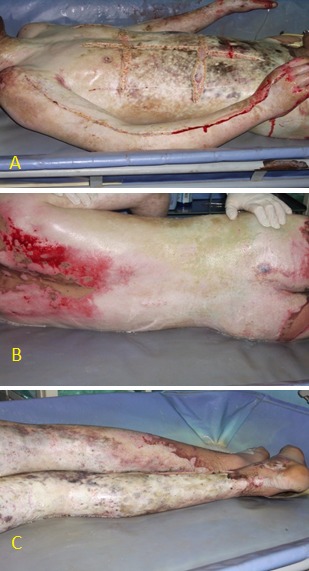
A young patient with a 95% TBSA burn

**Fig. 2:**
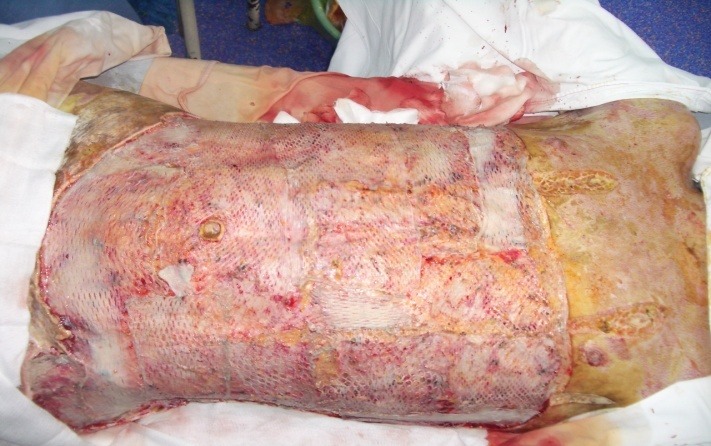
Sandwich graft of 6:1 autograft and 3:1 allograft

**Fig. 3:**
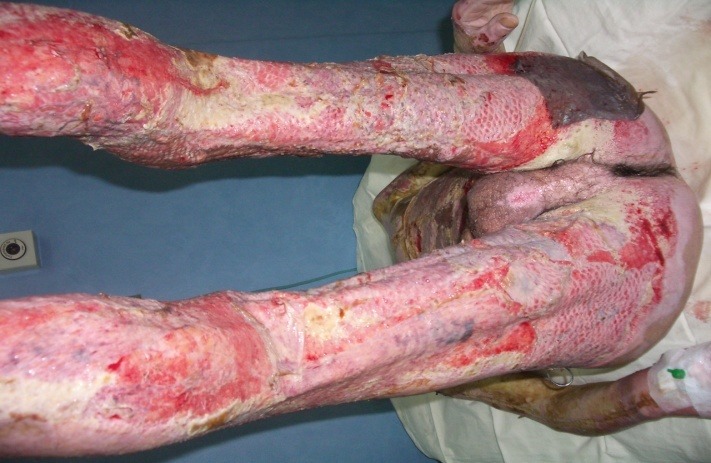
Allografts rejection, they needed to be replaced by autografts

**Fig. 4:**
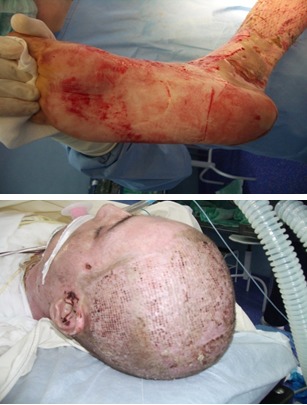
Autografts from plantar area and scalp

**Fig. 5:**
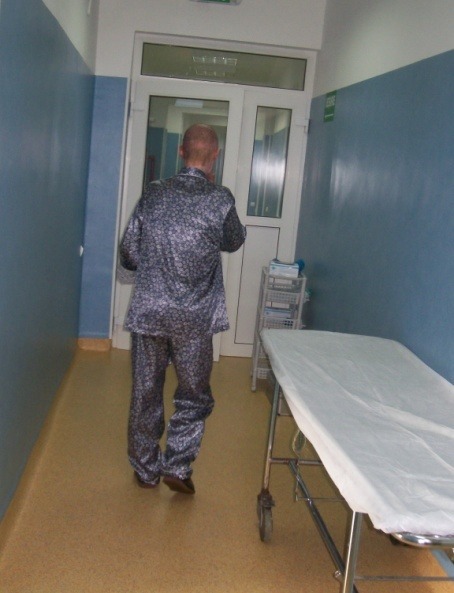
Day 69 – Discharge from the hospital

 Day 32- allografts rejection (needed to be replaced by autografts)

 He needed 6 surgical interventions, we took autografts from unusual sites like plantar area and scalp (three times harvesting for the scalp area).

On day 69, the patient was ready to be dismissed for ambulatory treatment.

## Discussion

Fresh or refrigerated allografts can tolerate modest wound contamination and adhere better to the freshly excised subcutaneous fat than cryopreserved grafts do. The allogeneic skin is usually removed once the patient’s donor sites are healed sufficiently for the re-harvesting or once autologous cultured skin is available for permanent wound closure. 

When refrigerated (fresh) allograft is not available, cryopreserved skin is an excellent alternative for temporary wound coverage. Although frozen cryopreserved skin has less measurable viability than fresh skin, it is generally difficult to maintain continuous and ample stores of fresh skin beyond 14 days [**5**].

The use of allograft can have potential disadvantages such as infection, rejection, etc.

Allograft skin has been reported to cause bacterial infection [**6**]. Although White [**7**] has suggested that cadaver allograft can be used for wound coverage, current standards [**8,9**] require that skin is discarded if pathogenic bacteria or fungi are present. This is particularly important given the immunocompromised status of the potential recipient has, an immunocompromised status that is likely to develop sepsis following such contamination. 

There have also been reports of viral disease transmission by skin allografts. In 1987, Clarke [**10**] reported the transmission of HIV-1 to a burn patient from an HIV-positive donor; results of donor testing were not known before skin use. Kealey [**11**] recently reported the transmission of cytomegalovirus (CMV) in cadaver skin allografts. 

Herndon and Rose [**12**] state that the benefits of donor allografts for the treatment of burn patients clearly outweigh the small risk.

Rejection 

Although demonstrating many characteristics of an ideal wound covering, allograft skin contains Langerhans cells that express class II antigens on their surface. These cells reside in the epidermis of the skin and will ultimately result in an immunologic rejection response. This typically results in an acute inflammatory reaction and may lead to the development of a deep wound infection. Vascularized allogeneic skin grafts typically remain intact on the wound of a burn patient for 2 to 3 weeks, although there have been reports of allograft skin survival for up to 67 days, due in part to the inherent immunosuppression of extensive burn injury [**13**]. Recent improvements in immunonutrition, critical care management, and a more aggressive surgical approach to definitive wound closure, however, have made the persistence of engraftment less predictable. 

Efforts to prevent rejection have included methods that might reduce antigen expression by controlling Langerhans cell activity in the allograft skin. Treatment of the allografts with ultraviolet light irradiation and incubation of the skin in glucocorticoids has been reported to result in a modest prolongation of allograft survival compared with untreated skin. 

There are published reports of the successful use of allograft with systemic immunosuppression that achieve wound closure [**3,4**]. This idea has not been widely accepted until now.

The effects of pharmacologic agents to induce immunosuppression in patients with major burn injuries [**14**] have been reported to improve allograft and patient survival in children treated with azathioprine and antithymocyte globulin, even though this was associated with azathioprine- induced neutropenia. Skin allograft survival in patients with extensive full-thickness thermal injuries has been prolonged by the use of Cyclosporine A [**15,16**]. Allograft rejection is generally observed within a few days from discontinuing treatment; however, there have been instances where engraftment has persisted after the completion of therapy.

## Conclusion

The indications for allograft skin use in wound management are the covering of the extensive wounds where autologous tissue is not available and the covering of widely meshed skin autografts.

 They can also be used is cases of extensive partial thickness burns or extensive epidermal slough. 

 By applying an allograft, the ability to accept an autograft and create a template for the delayed application of keratinocytes can be tested. 

 The advantages of human allograft skin use are that they reduce water, electrolyte, and protein loss, prevent desiccation of tissue, suppress bacterial proliferation, reduce wound pain, reduce energy requirements, promote epithelialisation, prepare wounds for definitive closure, and provide dermal template for epidermal grafts [**5**].

 The use of allografts in our clinic improved the overall life expectancy of majorly burned patients, so that we could hope that if all factors concur, the use of allografts will lead to a good outcome: 52 surviving patients treated with allografts from the first 148 who survived in the first period of time (2002-2009) also 9 from the 47 patients (2010-2011).

**Acknowledgements**

 "This paper is partially supported by the Sectoral Operational Programme of Human Resources Development, financed by the European Social Fund and by the Romanian Government under the contract number POSDRU/89/1.5/S/64153"
